# *Gynostemma pentaphyllum* Extract Alleviates NASH in Mice: Exploration of Inflammation and Gut Microbiota

**DOI:** 10.3390/nu16111782

**Published:** 2024-06-06

**Authors:** Feng-Yan Jiang, Si-Ran Yue, Yi-Yun Tan, Nan Tang, Yue-Song Xu, Bao-Jun Zhang, Yue-Jian Mao, Zheng-Sheng Xue, Ai-Ping Lu, Bao-Cheng Liu, Rui-Rui Wang

**Affiliations:** 1 Shanghai Innovation Center of TCM Health Service, Shanghai University of Traditional Chinese Medicine, Shanghai 201203, China; jiangfy_1101@163.com (F.-Y.J.); yuesiran@163.com (S.-R.Y.); yiyuntan111@163.com (Y.-Y.T.); nnantang@163.com (N.T.); xysong02@connect.hku.hk (Y.-S.X.); bjaida89@163.com (B.-J.Z.); 2 Li Ka Shing Faculty of Medicine, The University of Hong Kong, Pokfulam, Hong Kong 999077, China; 3 China Mengniu Dairy Company Limited, Hohhot 010000, China; maoyuejian@mengniu.cn (Y.-J.M.); xuezhengsheng@mengniu.cn (Z.-S.X.); 4 School of Chinese Medicine, Hong Kong Baptist University, Kowloon Tong, Hong Kong 999077, China; aipinglu@hkbu.edu.hk

**Keywords:** *Gynostemma pentaphyllum*, NASH, traditional Chinese medicine, intestinal microbiota, inflammatory

## Abstract

NASH (non-alcoholic steatohepatitis) is a severe liver disease characterized by hepatic chronic inflammation that can be associated with the gut microbiota. In this study, we explored the therapeutic effect of *Gynostemma pentaphyllum* extract (GPE), a Chinese herbal extract, on methionine- and choline-deficient (MCD) diet-induced NASH mice. Based on the peak area, the top ten compounds in GPE were hydroxylinolenic acid, rutin, hydroxylinoleic acid, vanillic acid, methyl vanillate, quercetin, pheophorbide A, protocatechuic acid, aurantiamide acetate, and iso-rhamnetin. We found that four weeks of GPE treatment alleviated hepatic confluent zone inflammation, hepatocyte lipid accumulation, and lipid peroxidation in the mouse model. According to the 16S rRNA gene V3–V4 region sequencing of the colonic contents, the gut microbiota structure of the mice was significantly changed after GPE supplementation. Especially, GPE enriched the abundance of potentially beneficial bacteria such as *Akkerrmansia* and decreased the abundance of opportunistic pathogens such as *Klebsiella*. Moreover, RNA sequencing revealed that the GPE group showed an anti-inflammatory liver characterized by the repression of the NF-kappa B signaling pathway compared with the MCD group. Ingenuity Pathway Analysis (IPA) also showed that GPE downregulated the pathogen-induced cytokine storm pathway, which was associated with inflammation. A high dose of GPE (HGPE) significantly downregulated the expression levels of the tumor necrosis factor-α (TNF-α), myeloid differentiation factor 88 (Myd88), cluster of differentiation 14 (CD14), and Toll-like receptor 4 (TLR4) genes, as verified by real-time quantitative PCR (RT-qPCR). Our results suggested that the therapeutic potential of GPE for NASH mice may be related to improvements in the intestinal microenvironment and a reduction in liver inflammation.

## 1. Introduction

The spectrum of non-alcoholic fatty liver disease (NAFLD) encompasses two principal subtypes: non-alcoholic fatty liver (NAFL) and non-alcoholic steatohepatitis (NASH) [1]. However, NASH is the more severe subtype, which is characterized by hepatocellular lipid accumulation with inflammatory infiltration and fibrosis [2]. NASH could lead to hepatocellular carcinoma (HCC), and the annual incidence of HCC in NASH cirrhosis patients is estimated to range from 0.5% to 2.6% [3]. In addition, NASH is considered to be the second most common indication for liver transplantation in the US, following chronic hepatitis C [4]. In March 2024, the FDA approved the first innovative drug, which aimed at adult patients with NASH accompanied by liver fibrosis [5]. Before this, the management of NASH primarily focused on lifestyle intervention, including dietary changes and physical activity, alongside the management of associated comorbidities such as metabolic disorder and sarcopenia [6,7,8].

There are trillions of gut bacteria in the human gut, and symbiotic bacteria are closely related to host metabolism [9,10]. Gordon et al. discovered initial evidence linking gut microecological dysbiosis to NAFLD, showing that lean rodents developed metabolic changes that were identical to obese rodents after receiving their gut microbiota [11]. Recent research showed that *Bacteroides stercorisa*, a specific bacterium, can accelerate the progression of NAFLD [12]. The liver–gut axis facilitates liver injury by exposing it to gut-derived bacteria and their components [13]. Bacterial components could enter the portal vein and access the liver, promoting hepatic inflammation through Kuffer cell activation by Toll-like receptor 4 (TLR4) [14]. Moreover, it has been reported that many natural drugs can reshape the intestinal microbiota [15].

Chinese herbal medicine is widely used for the prevention and treatment of NAFLD due to its efficacy and minimal side effects [16]. A large number of active components in Traditional Chinese Medicine (TCM), such as saponins, polysaccharides, and flavonoids, have shown efficacy in targeting the gut microbiota to treat NASH [17,18,19]. The saponin extract of *Polygala japonica* Houtt ameliorated NASH in mice by restoring the gut microbiota balance and affecting the metabolites in their feces and liver [20]. MDG-1, an Ophiopogon polysaccharide, promotes short-chain fatty acid production in the intestine, thereby regulating inflammatory responses and lipid metabolism in the liver [21]. Tetrastigma hemsleyanum leaves’ extracts, which are rich in flavonoids and phenolic acids, ameliorate NASH by protecting the gut barrier and increasing the relative abundance of beneficial bacteria [22]. *Gynostemma pentaphyllum* (Thunb.) Makino (GP) stands out as a prevalent and potent herbal medicine in treating liver-related metabolic disease [23]. Meanwhile, it is also a tea favored for its pleasant taste and effectiveness in weight loss. GP, as a typical edible and medicinal plant, was officially classified as a functional food by the Chinese Ministry of Public Health on 5 March 2002 [24]. As a highly promising natural product, GP has attracted significant attention for its multiple effective biological activities, including immune activity modulation, anti-tumor activity, liver protection, and neuroprotection [25,26,27,28]. Furthermore, GPE, which is rich in gypenosides, has been shown to mitigate NAFLD by regulating the hepatic lipid metabolism and reducing inflammation [29,30]. However, there is limited pharmacological research on *Gynostemma pentaphyllum* extract (GPE), which is rich in flavonoids and fatty acids. Hence, the potential of GPE to target the gut microbiota to alleviate hepatic inflammation and the specific mechanisms of this need further exploration.

In this study, we investigated the anti-inflammatory effects of GPE on methionine–choline-deficient (MCD) diet-induced NASH in mice and revealed the protective effects in the liver and the underlying mechanisms of GPE via transcriptomics and intestinal microbiomics.

## 2. Materials and Methods

### 2.1. Preparation of GPE

GP decoction pieces were extracted in 80 °C water for 2 h (GP decoction pieces:water = 1:20; volume ratios). The crude extracts were filtered through an AB-8 macroporous adsorption resin column and then eluted with water. A freeze-dryer was used to freeze-dry the extract for 48 h. After that, 75% alcohol was used to elute the extract. The ethanol extract was concentrated at 45 °C with a vacuum rotary evaporator and then put in a vacuum drying oven to dry at 45 °C for more than 12 h to obtain GPE. 

### 2.2. Compositional Identification of GPE 

The mass spectrometry data for the GPE sample were collected by ultra-performance liquid chromatography-quadrupole time-of-flight mass spectrometry (UPLC-Q-TOF-MS). Data acquisition was conducted using Analyst TF 1.7.1 software. The processing of the data was carried out using Peakview 1.2. Identification was prioritized by matching the mass spectrometry data with the Natural Products HR-MS/MS Spectral Library 1.0 database, and the compounds were preliminarily screened based on the score information of each peak and further confirmed based on the first-order and second-order information of each peak. Sample preparation involved taking the necessary quantity of the sample and adding 2 ml of methanol. The mixture was then subjected to ultrasonic dissolution and passed through a microporous filter membrane. The chromatographic conditions were as follows: chromatographic column: Agilent ZORBAX RRHD SB-C18 (2.1 × 100 mm, 1.8 µm, Santa Clara, CA, USA); column temperature: 30 °C; flow speed: 0.3 mL/min; input sampling: 2 μL; detection of wavelengths: 190~400 nm; and mobile phase ratio: phase A acetonitrile and phase B aqueous solution. The gradients are shown in Table 1. The mass spectrometry conditions were as follows: mass spectrometry detection mode: ESI-negative/positive ion mode; the mass parameters are shown in Table S2. The instruments used in the experiment were: a Waters H-Class UPLC system (Waters, Milford, MA, USA); an AB Sciex Triple TOF^®^ 4600 system (AB SCIEX, Framingham, MA, USA); an analytical balance (#ME104, METTLER TOLEDO, Shanghai, China); an ultrasonic cleaner (#KQ-300 BD, Kun Shan Ultrasonic Instruments Co., Ltd., Kunshan, China); and a centrifugal machine (#SIGMA 3K15, SIGMA, Osterode am harz, Germany). The gradient profile of the mobile phase and the mass spectrometry parameters are detailed in Tables S2 and S3.

### 2.3. Animal Studies and Intervention

Nine-week-old male C57BL/6J mice were purchased from Shanghai Model Organisms Center (Shanghai, China) and fed until they were 10 weeks old. After 1 week of acclimatization, the 28 mice were randomly divided into four groups, with each group comprising 7 mice. All mice were housed in a 12 h light (7 a.m. to 7 p.m.) and 12 h dark (7 p.m. to 7 a.m.) cycle with free access to water and a chow diet, and all interventions were given by oral gavage.

The control group was fed with an MCS (#519581, Dyets, Bethlehem, PA, USA) diet, and the model group was fed with an MCD (#519580, Dyets, Bethlehem, PA, USA) diet for 4 weeks. In the LGPE group, the mice were fed with an MCD diet and low doses of GPE (150mg/kg). In the HGPE group, the mice were fed with an MCD diet and high doses of GPE (300mg/kg). After 4 weeks, all mice were fasted overnight and sacrificed for sampling. For long-term storage, all samples were stored at −80 °C. 

### 2.4. Histopathological Evaluation

For the liver pathology evaluation, we performed different staining treatments on the liver sections. The liver tissue was fixed with 4% paraformaldehyde and then embedded in paraffin for sectioning. The sections were stained with Hematoxylin and Eosin (H&E), Oil Red O, and Sirius Red. The Non-Alcoholic Fatty Liver Disease Activity Score (NAS) was utilized, which evaluates the severity of NASH based on the degree of steatosis, periportal inflammation, and hepatocellular ballooning [31]. The results from the H&E staining and Oil Red O staining were utilized to calculate the statistical NAS score. Sirius-Red-stained sections were employed to quantify the collagen fiber area, thereby assessing the extent of liver fibrosis. Images were acquired and analyzed using ImageJ (1.53a).

### 2.5. Serum and Liver Biochemical Assays

The levels of hepatic total cholesterol (TC, #A111-1-1), triglycerides (TG, #A110-1-1), superoxide dismutase (SOD, #A001-3-2), malondialdehyde (MDA, #A003-1-2), and hydroxyproline (HYP, #A030-2-1) were quantitatively measured using assay kits from Nanjing Jiancheng Bioengineering Institute. The blood indicators, including low-density lipoprotein cholesterol (LDL-C), high-density lipoprotein cholesterol (HDL-C), alanine transaminase (ALT), aspartate transaminase (AST), TC, and TG, were measured at the Center for Drug Safety Evaluation and Research, Shanghai University of Traditional Chinese Medicine. Detailed experimental methods are provided in the kit’s instructions, and the experimental procedures were rigorously adhered to.

### 2.6. Gut Microbiome 16S rRNA Gene Sequencing

To further investigate the structural and functional characteristics of the intestinal microbiota, we used high-throughput techniques to sequence the V3-V4 variable region of the 16S ribosomal RNA (rRNA) of the gut microbiota in the mouse colon contents; this analysis was conducted by HonsunBio Biotechnology Co., Ltd. (Shanghai, China). The genomic DNA of the gut microbiota was extracted using the E.Z.N.A ^®^ Stool DNA Kit (#D4015-04, Omega Biotek, Norcross, GA, USA). The purity and concentration of the extracted DNA were determined with a NanoDrop spectrophotometer (ThermoFisher, Waltham, MA, USA). The quality of the extracted DNA was checked on a 1% agarose gel. The V3–V4 regions of the 16S rRNA gene were amplified using 338F (5′-ACTCCTACGGGAGGCAGCAG-3′) and 806R (5′-GGACTACHVGGGTWTCTAAT-3′) primer pairs. The purity of the product was determined using the AxyPrep DNA Gel Extraction Kit (#AP-GX-250G, Axygen Biosciences, Union City, CA, USA). The quality of the product was measured by a QuantusTM Fluorometer (Promega, Madison, WI, USA). Amplicon sequencing was performed using the Illumina MiSeq PE300 system (Promega, San Diego, CA, USA). Raw data files were quality-filtered and merged using FLASH. The reads were clustered into operational taxonomic units (OTUs) with a 97% similarity cutoff using Usearch and aligned using the SILVA database. 

### 2.7. Hepatic Transcriptome

Transcriptome RNA sequencing (RNA-Seq) was performed using Illumina high-throughput RNA sequencing. Three samples were selected from each group. The total RNA was extracted using Trizol Reagent (#15596018, Austin, TX, USA). The RNA Seq library was constructed using the TruSeq RNA Sample Preparation Kit (Illumina, San Diego, CA, USA), and sequencing was performed on a Hiseq platform (Illumina) by Shanghai Personal Biotechnology Cp. Ltd. (Shanghai, China). After sequencing, the reads were preprocessed based on Cutadapt with an average quality score lower than Q20 and filtered. A differential expression analysis was performed using DESeq (1.30.0). DEGs with a *p* < 0.05 and a |log2 fold change| > 1 were considered to be significantly differentially expressed genes. The data were analyzed on the online platform of the Personalbio Genes Cloud Platform (www.genescloud.cn/login, accessed on 3 December 2020).

### 2.8. Real-Time Quantitative PCR

The total RNA was extracted with a FastPure Cell/Tissue Total RNA Isolation Kit V2 (#RC112, vazyme, Nanjing, China) from the liver tissues. cDNA was synthesized by the Hiscript III RT SuperMix for qPCR (+gDNA wiper) (#R223, Vazyme, Nanjing, China). Then, quantitative real-time PCR (RT-qPCR) was carried out using the Luna Universal qPCR Master Mix (#M3003M, New England Biolabs, Inc., Ipswich, MA, USA). The experimental methods are detailed in the instructions of the reagent box, and the experimental steps were strictly followed. The primers were synthesized by Sangon Biotech (Shanghai) Co., Ltd. (Shanghai, China) (Table S4). Glyceraldehyde 3-phosphate dehydrogenase (GAPDH) is a housekeeping gene. Relative quantification was calculated using the 2^−ΔΔCt^ method.

### 2.9. Assay for IL-1β and LBP

The serum interleukin-1 beta (IL-1β) and lipopolysaccharide-binding protein (LBP) levels of the MCS, MCD, LGPE, and HGPE groups were detected using a mouse IL-1β ELISA kit (#PCDBM0158, Shanghai Panchao Biotechnology Co., Ltd., Shanghai, China) and a mouse LBP ELISA kit (#PCDBN0177, Shanghai Panchao Biotechnology Co., Ltd., Shanghai, China). The experimental methods are detailed in the instructions of the reagent box, and the experimental steps were strictly followed.

### 2.10. Statistical Analysis

All data are represented as the mean ± standard error of the mean (SEM). Most of the plots were generated using GraphPad Prism 9.5.0 (GraphPad Software, San Diego, CA, USA). All statistical analyses were performed using SPSS 26.0 software (Chicago, IL, USA). Comparisons between the two groups were conducted using either the *t*-test or the Mann–Whitney U test. Comparisons among multiple groups were conducted using either one-way analysis of variance (ANOVA), least significant difference (LSD), or the Kruskal–Wallis test. When the data conformed to normality and had a homogeneity of variance, *t*-tests and one-way ANOVA LSD were used. Otherwise, the Mann–Whitney U test and Kruskal–Wallis test were employed. A *p* < 0.05 was considered to be statistically significant.

## 3. Results

### 3.1. The Analysis of GPE Compositions

**Figure 1:**
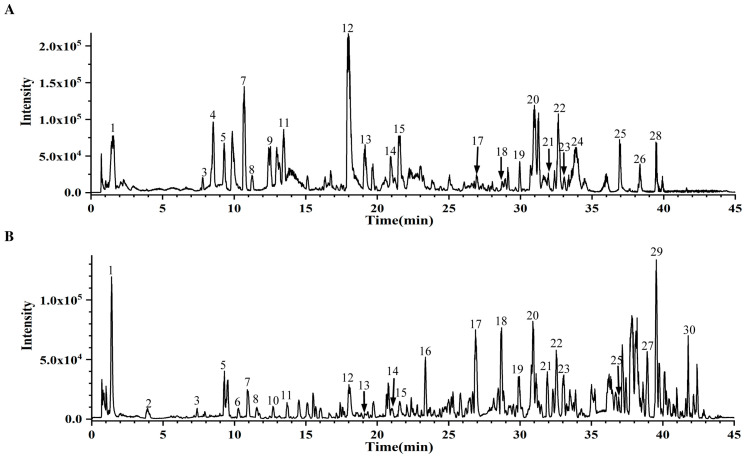
Analysis of GPE compositions. Base peak chromatogram (BPC) of GPE in the negative (**A**) and positive (**B**) ion modes.

We utilized ultra-performance liquid chromatography quadrupole time-of-flight mass spectrometry (UPLC-Q-TOF/MS) technology to analyze the active components of GPE. We identified 30 compounds by correlating the fragment ion peak data and retention times with the Natural Products HR-MS/MS Spectral Library 1.0 database (Figure 1, Table S1). The top 10 relative abundances compounds were identified as hydroxylinolenic acid, rutin, hydroxylinoleic acid, Vanillic acid, methyl vanillate, quercetin, pheophorbide A, protocatechuic acid, aurantiamide acetate, and iso-rhamnetin (Table 1). Among these, the composition encompasses two flavonoids, two fatty acids, two phenolic acids, one flavonoid glycoside, one phenol, one dipeptide, and one chlorophyll-related compound.

### 3.2. GPE Attenuates Liver Injury and Oxidative Stress in NASH Mice

**Figure 2:**
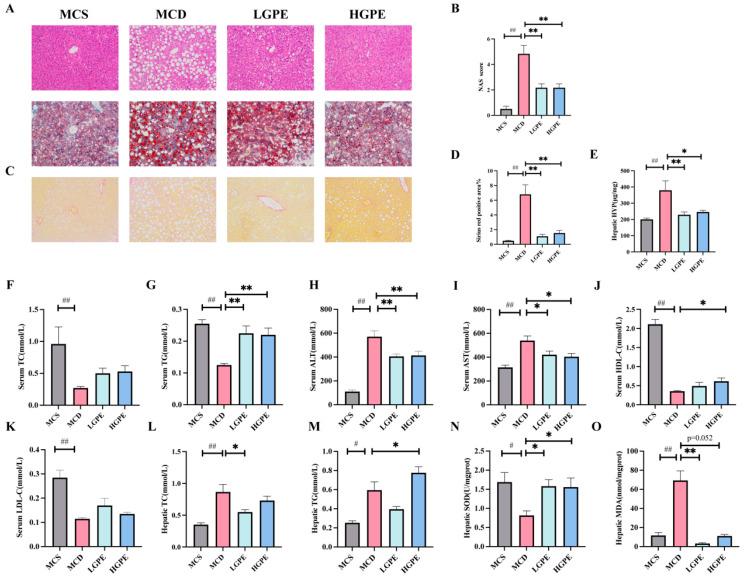
GPE ameliorates liver injury in MCD diet-induced NASH mice. (**A**) H&E-staining (200 magnifications, 100 μM) and Oil Red (200 magnifications, 100 μM) of liver sections; (**B**) Non-alcoholic Fatty Liver Disease Activity Score (NAS); (**C**) Sirius Red staining (200 magnifications, 100 μM) of liver sections; (**D**) quantification of the percentage of the Sirius-Red-positive area; (**E**) hepatic hydroxyproline quantification (HYP); (**F**–**M**) serum total cholesterol (TC), serum triglycerides (TG), serum alanine aminotransferase (ALT), serum aspartate aminotransferase (AST), serum high-density lipoprotein cholesterol (HDL-C), serum low-density lipoprotein cholesterol (LDL-C), hepatic TC, and hepatic TG; and (**N**,**O**) hepatic superoxide dismutase and malondialdehyde quantification (SOD and MDA). Each biological replicate (n = 5–6) had three technical replicate wells for the experiment. Data are expressed as means ± SEM. # *p* < 0.05, ## *p* < 0.01 vs. the MCS group; * *p* < 0.05, ** *p* < 0.01 vs. the MCD group.

To investigate the pharmacological effects of GPE on liver steatosis, hepatocyte inflammation, and other related damage, we used an MCD-induced NASH mouse model that caused liver inflammation and fibrosis in addition to simple steatohepatitis, where the observed association between the activation of Kupffer cells and liver pathological features reflected the human NASH etiology (Figure 2A). Hematoxylin and eosin (H&E) and oil red O staining showed that hepatocyte ballooning in hepatocytes and inflammation of the sink area in the MCD-induced NASH mice were significantly reduced by GPE (Figure 2B,C). In addition, the Sirius Red staining and hepatic Hydroxyproline (HYP) results indicated that GPE reduced the area of collagen fibers (Figure 2D,E). Compared with the MCS group, serum total cholesterol (TC), triglycerides (TG), high-density lipoprotein cholesterol (HDL-C), and low-density lipoprotein cholesterol (LDL-C) (*p* < 0.01) were significantly decreased and serum alanine aminotransferase (ALT) and aspartate aminotransferase (AST) (*p* < 0.01) were significantly increased in the MCD group; compared with the MCD group, serum TG (*p* < 0.01) was significantly increased and ALT (*p* < 0.01) and AST (*p* < 0.05) were significantly decreased in the LGPE group; and compared with the MCD group, TG (*p* < 0.01) and HDL-C (*p* < 0.05) were significantly increased and ALT (*p* < 0.01) and AST (*p* < 0.05) were significantly decreased in the HGPE group (Figure 2F–K). Additionally, compared with the MCS group, hepatic TC (*p* < 0.01), TG (*p* < 0.05), and malondialdehyde (MDA) were significantly increased and superoxide dismutase (SOD) was significantly decreased in the MCD group; compared with the MCD group, hepatic TC (*p* < 0.05) and MDA (*p* < 0.01) were significantly decreased and SOD (*p* < 0.05) was significantly increased in the LGPE group; and compared with the MCD group, hepatic TG and SOD (*p* < 0.05) were significantly increased in the LGPE group (Figure 2L–O). The above results suggest that both LGPE and HGPE can partially improve liver fibrosis, hepatocyte fat accumulation, and peroxidative damage in MCD diet-induced NASH mice to further protect the liver.

### 3.3. GPE Divergently Alters the Structure of Gut Microbiota

**Figure 3:**
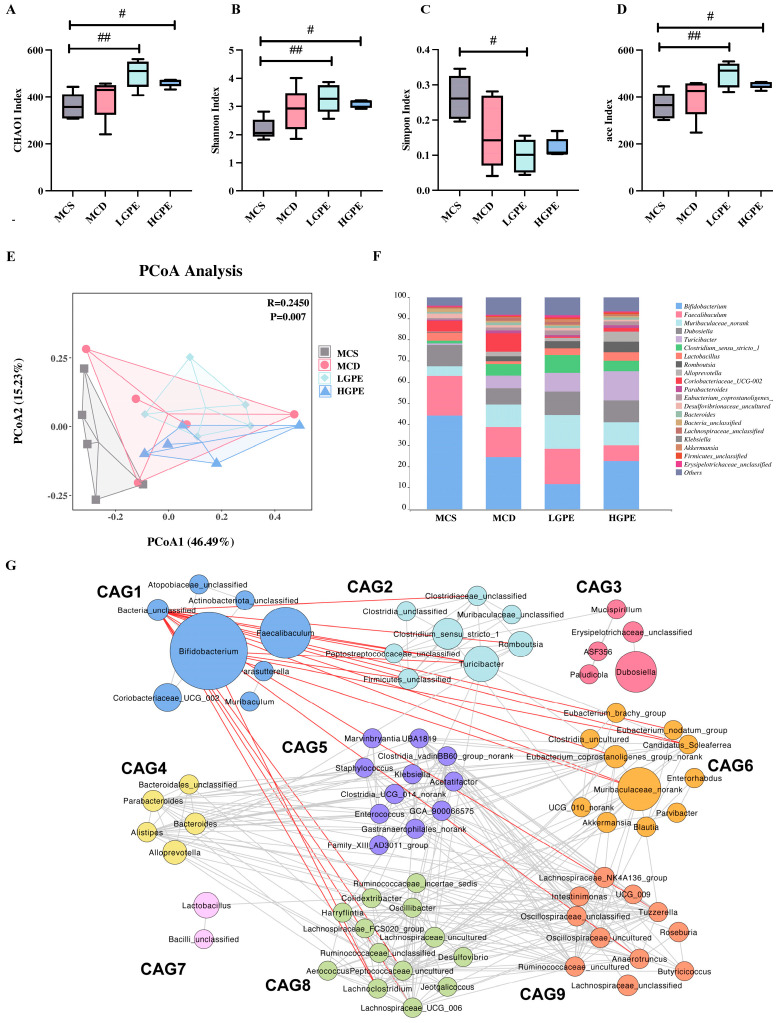
GPE alters the structure of the intestinal microbiota. (**A**–**D**) The alpha diversity analysis of sequence reads. (**A**) Chao1 index. (**B**) Shannon index. (**C**) Simpson. (**D**) ACE index. (**E**) Principal co-ordinate analysis at the OTU level based on the Bray–Curtis distance. (**F**) Fecal microbiota at the genus level. (**G**) Co-abundance groups interaction network of genus level among all groups based on Pearson and Spearman correlation statistical analysis. The network shows correlation relationships between nine CAGs of 174 genera. Node size represents the average abundance of each genus. Lines between nodes represent correlations of each other, with the line width representing the correlation magnitude. The red ones represent positive correlations, and the blue ones represent negative correlations. Data are expressed as means ± SEM. # *p* < 0.05, ## *p* < 0.01 vs. the MCS group.

The composition of the intestinal microbiota can be determined by sequencing the V3-V4 region of the 16S rRNA gene, enabling an analysis of the alterations in the microbiota structure due to GPE treatment. The α-diversity of the microbiota assessed from the CHAO1, Shannon, Simpson, and ace indices showed that there was a significant difference between MCS and LGPE/HGPE but not between MCS and MCD (Figure 3A–D). In addition, a principal coordinate analysis (PCoA) and PERMANOVA based on Bray–Curtis distance showed that the gut microbiota structure was significantly different among the four groups (Figure 3E). We ranked the top 20 genera in each of the four groups, noting that LGPE and HGPE regulated the structure of the intestinal microbiota (Figure 3E). Most strikingly, the proportion of the *Akkermansia* genus increased from 0.15% to 1.35% after LGPE intervention. As the bacteria act as interdependent functional groups in the gut ecosystem, we constructed a co-abundance network in which the 174 genera were shared by at least 20% of the samples based on a Pearson correlation statistical analysis and clustered the genus into nine co-abundance group CAGs (Figure 3G and Supplementary Figure S1). The results showed that the relative abundance of the seven CAGs had no significant difference among the four groups. The relative abundance of CAG1 was significantly lower in the LGPE and HGPE groups than in the MCS group. The relative abundance of CAG2 was significantly higher in the HGPE group than in the MCS group. Moreover, CAG1 included eight operational taxonomic units (OTUs), mainly from the genera *Bifidobacterium*, *Faecalibaculum*, and *Coriobacteriaceae_UCG_002*; CAG2 included eight OTUs, mainly from the genera *Turicibacter*, *Clostridium_sensu_stricto_1*, and *Peptostreptococcaceae_unclassified*. Additionally, the relative abundance of CAG5 was higher in the MCD group and showed a decreasing trend in the LGPE and HGPE groups, in which *Klebsiella* was the main genus of CAG5. The relative abundance of CAG6 was lower in the MCS group and showed an increasing trend in the LGPE and HGPE groups, in which *Akkermansia* was the main genus. Similarly, the relative abundance of CAG7 was lower in the MCD group and showed an increasing trend in the LGPE and HGPE groups, in which *Lactobacillus* was the main genus.

### 3.4. GPE Enriches Potential Probiotics and Reduces Opportunistic Pathogens

**Figure 4:**
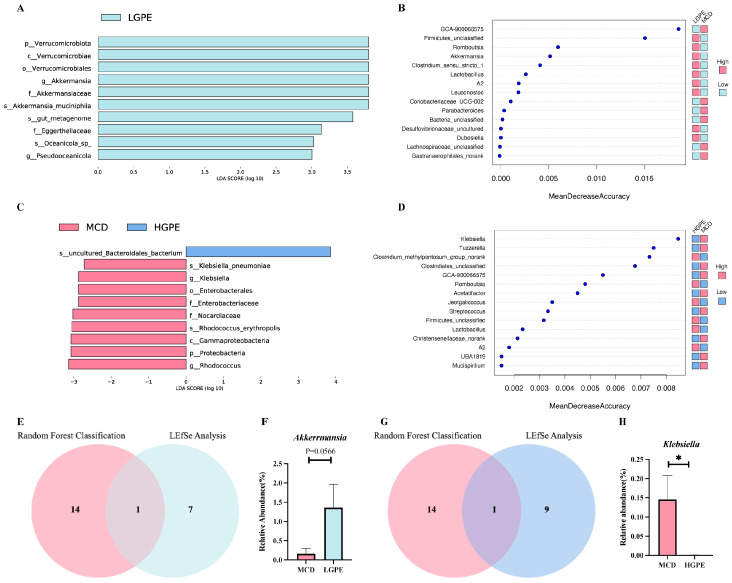
GPE enriches potential probiotics and reduces opportunistic pathogens. (**A**) Linear discriminant analysis (LDA) effect size (LEfSe) analysis for differences in abundance between MCD and LGPE groups of biomarkers; (**B**) random forest classification for top 15 bacterial genera between MCD and LGPE groups; (**C**) linear discriminant analysis (LDA) effect size (LEfSe) analysis for differences in abundance between MCD group and HGPE groups of biomarkers; (**D**) random forest classification for top 15 bacterial genera between MCD group and HGPE groups; (**E**) Venn diagrams to show the common biomarker for LEfSe analysis and random forest classification between MCD and LGPE groups; (**F**) the relative abundance of *Akkermansia* between MCD and LGPE groups; (**G**) Venn diagrams to show the common biomarker for LEfSe analysis and random forest classification between MCD group and HGPE groups; and (**H**) the relative abundance of *Klesiella* between the MCD and LGPE groups. Data are expressed as means ± SEM. * *p* < 0.05 vs. the MCD group.

The linear discriminant analysis effect size (LEfSe) and random forest analysis were utilized to identify biomarkers that effectively differentiated between the modeling and medication groups. The LEfSe analysis between the MCD and LGPE groups showed that *Akkermansia* and *Pseudooceanicola* were enriched in the LGPE group (Figure 4A). However, in the random forest analysis, the top 10 genera with the most significant differential contributions between the MCD and LGPE groups included *GCA-900066575*, *Firmicutes_unclassified*, *Romboutsia*, *Akkermansia*, *Clostridium_sensu_stricto_1*, *lactobacillus*, *A2*, *Leuconostoc*, *Coriobacteriaceae_UCG-002*, and *Parabacteroides* (Figure 4B). The LEfSe analysis between the MCD and HGPE groups showed that *Klebsiella* and *Rhodococcus* were enriched in the MCD group (Figure 4C). In the random forest analysis, the top 10 genera that contributed the most significantly to the prediction accuracy included *Klebsiella*, *Tuzzerella*, *Clostridium_methylpentosum_group_norank*, *Clostridiales_unclassified*, *GCA-900066575, Romboutsia, Acetatifactor, Jeotgalicoccus, Streptococcus,* and *Firmicutes_unclassified*, with the highest contributions to prediction accuracy (Figure 4D). This indicated that *Akkermansia* might have been a biomarker between the MCD and LGPE groups since it was a significant contributor in both the Lefse analysis and random forest analysis (Figure 4E). Further, we found that the relative abundance of *Akkermansia* showed an increased tendency in the LGPE group compared to the MCD group (Figure 4F). Similarly, we showed that the relative abundance of the biomarker *Klebsiella* was significantly decreased in the HGPE group compared to the MCD group (Figure 4G,H).

### 3.5. GPE-Regulated Hepatic Gene Expression Profile in MCD-Fed Mice

**Figure 5:**
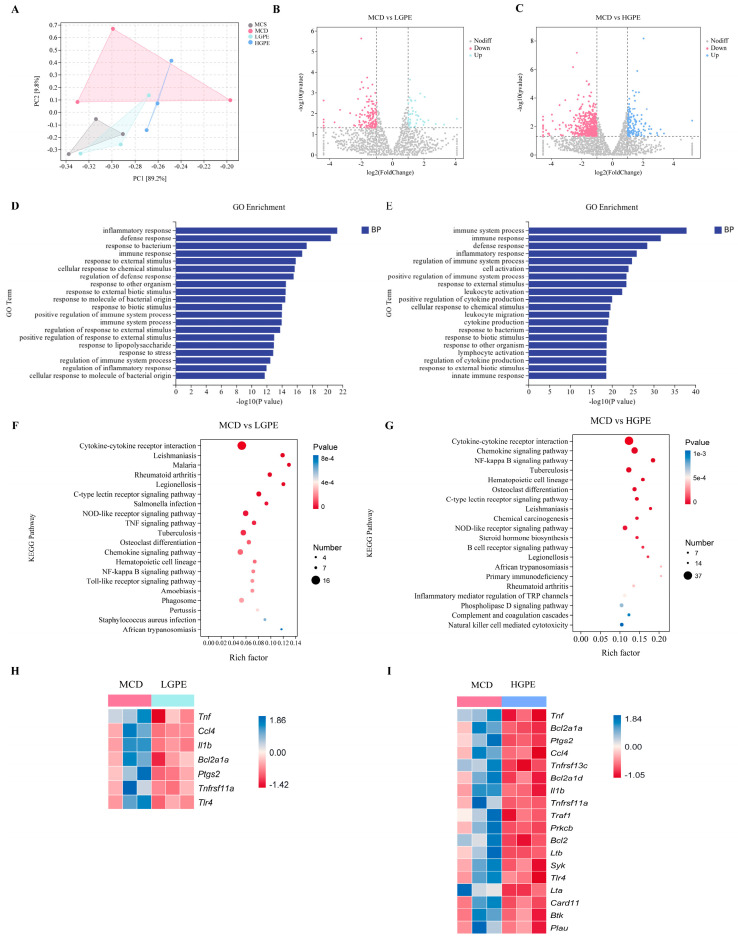
GPE-induced alterations in the liver transcriptome. (**A**) Principal component analysis (PCA) analysis of the transcript; (**B**,**C**) volcano plots show the differentially expressed genes of MCD vs. LGPE and MCD vs. HGPE, as revealed by the transcriptome; (**D**–**G**) Gene Ontology (GO) enrichment analysis of the top 20 pathways between the MCD and LGPE groups (**D**) Gene Ontology (GO) enrichment analysis of the top 20 pathways between the MCD and HGPE groups; (**E**) Kyoto Encyclopedia of Genes and Genomes (KEGG) enrichment analysis of the top 20 pathways between the MCD and LGPE groups; (**F**) Kyoto Encyclopedia of Genes and Genomes (KEGG) enrichment analysis of the top 20 pathways between the MCD and HGPE groups; and (**H**,**I**) heatmap of differential gene expression in the NF-kappa B signaling pathway between the MCD and LGPE groups and between the MCD and HGPE groups.

To further investigate the alterations in hepatic gene expression caused by the GPE treatment and its anti-NASH mechanism, we performed hepatic eukaryotic transcriptome assays on the livers of the different groups of mice. The differentially expressed genes (DEGs) were determined with the criteria of *p* < 0.05 and a fold change of ≥2. Principal component analysis (PCA) showed that the four groups were separated by distance, and the two treatment groups were closest to the control group at the PC1 (89.2%) level (Figure 5A). According to the volcano plots, the total number of differentially expressed genes in the MCD group compared to the LGPE group was 190. The LGPE group upregulated 43 differentially expressed genes and downregulated 147 differentially expressed genes (Figure 5B). Meanwhile, the total number of differentially expressed genes in the MCD group compared to the HGPE group was 673. The HGPE group upregulated 151 differentially expressed genes and downregulated 522 differentially expressed genes (Figure 5C). Next, the functions of the differentially expressed genes (DEGs) sets were examined by classifying them into functional categories based on Gene Ontology (GO) terms and the Kyoto Encyclopedia of Genes and Genomes (KEGG) pathways. Based on the GO analysis, the DEG sets under the LGPE administration were predominantly enriched in pathways involved in the inflammatory response, defense response, and response to bacteria, and the DEG sets under HGPE were mainly enriched in pathways related to the immune system process, immune response, and defense response (Figure 5E,F). In addition, based on the KEGG analysis, pathways associated with inflammation were enriched under the LGPE administration (Figure 5F), and pathways involved in cytokine–cytokine receptor interactions, the chemokine signaling pathway, and the NF-kappa B signaling pathway were enriched under the HGPE administration (Figure 5G). Consistently, both LGPE and HGPE were simultaneously enriched with the NF-kappa B signaling pathway. Notably, Tnf, Ccl4, 1L-1β, Bcl2a1a, Ptgs2, Tnfrs11a, and Tlr4, which are related to inflammation, were commonly identified under the two treatments (Figure 5H,I).

### 3.6. Enriched Biological Pathways by IPA Analysis

**Figure 6:**
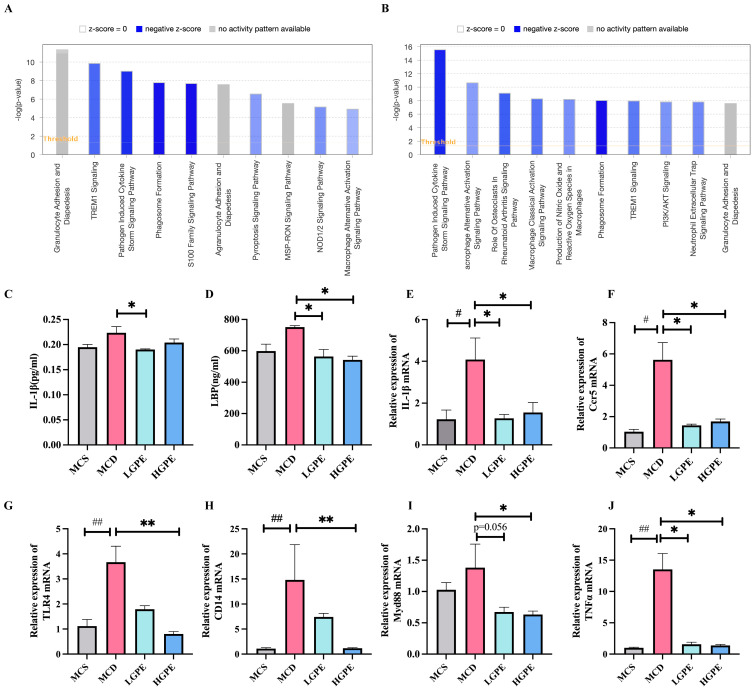
GPE suppresses the expression of inflammatory genes in the liver. (**A**) Pathway analysis of liver tissue of NASH mice based on transcriptomics in combination with ingenuity pathway analysis (IPA) between the MCD and LGPE groups; (**B**) pathway analysis of liver tissue of NASH mice based on transcriptomics in combination with ingenuity pathway analysis (IPA) between the MCD and HGPE groups; (**C**) the level of IL-1β in blood serum was tested by enzyme-linked immunosorbent assay (ELISA) (n = 4); (**D**) the level of LBP in blood serum was tested by ELISA (n = 4); (**E**–**J**) RT-PCR of IL-1β, Ccr5, TLR4, CD14, Myd88, and TNF-α mRNA expression in liver. The relative expression of IL-1β, Ccr5, TNF-α, Myd88, CD14, and TLR4 was adjusted with GAPDH as the housekeeping gene (n = 5). Each biological replicate (n = 4–5) had three technical replicate wells for the experiment. Data are expressed as means ± SEM. # *p* < 0.05, ## *p* < 0.01 vs. the MCS group; * *p* < 0.05, ** *p* < 0.01 vs. the MCD group.

Using the QIAGEN IPA bioinformatics tool, we found that pathogen-induced cytokine storm signaling pathway were down-regulated after GPE treatment (Figure 6A,B). Further RT-qPCR revealed that the gene expression of IL-1β (*p* < 0.05), Ccr5 (*p* < 0.05), TLR4 (*p* < 0.01), CD14 (*p* < 0.01), Myd88 (*p* < 0.05), and TNF-α (*p* < 0.05), which was enriched in the NF-κB signaling pathway, was significantly downregulated in the HGPE compared to the MCD group (Figure 6E–J). The expression of IL-1β (*p* < 0.05), Ccr5 (*p* < 0.05), and TNF-α (*p* < 0.05) were significantly downregulated in the LGPE compared to the MCD group (Figure 6E,F,J). We further investigated the serum levels of Interleukin-1 beta (IL-1β) and lipopolysaccharide binding protein (LBP); LGPE could significantly reduce IL-1β (*p* < 0.05) compared to the MCD group (Figure 6C). Both LGPE and HGPE could significantly reduce LBP (*p* < 0.05) compared to the MCD group (Figure 6D).

## 4. Discussion

NASH is a progressive form of NAFLD and is characterized by liver inflammation, hepatocellular steatosis, and injury [32,33]. This study revealed that GPE attenuated liver injury and oxidative stress in NASH mice. In addition, an enrichment of the potential probiotic *Akkermansia* was observed alongside a suppression of the opportunistic pathogen *Klebsiella* after GPE administration. GPE treatment also resulted in the reduced expression of inflammation-associated genes and the inhibition of inflammation-related pathways, underscoring its efficacy in ameliorating liver inflammation and modulating the intestinal microbiome in NASH mice.

The GPE treatment alleviated the symptoms of NASH in this study. Some studies have shown that GPE can alleviate the NASH-related comorbidity sarcopenia, which is mainly characterized by weakness, fatigue, and energy loss [7,34,35]. It was reported that GYP LXXV, a GP saponin-type extract, exerted its hepatoprotective effects by ameliorating hepatic lipid accumulation and hepatic fibrosis in NASH mice [30]. The absence of gypenoside in GPE may be related to the extraction technique and the source of natural medicine. GPE containing different components exhibits varying therapeutic effects. In the current study, GPE mainly comprised flavonoids, fatty acids, phenolic acids, and phenolics. Rutin, quercetin, isorhamnetin, and kaempferol are flavonoids. According to other studies, these compounds protect the liver by reducing hepatic adiposity [36,37,38,39]. Among them, the content of fatty acids is relatively high, including linolenic acid and linoleic acid, as well as hydroxylinolenic acid and hydroxylinoleic acid. The position of hydroxylation in fatty acids is closely related to their properties. Research has shown that 13-hydroxylinoleic acid has the effect of lowering blood pressure in rats [40]. Apparently, research on linoleic acid is more extensive. It has been demonstrated that moderate fatty acid intake reduces hepatic lipid accumulation [41,42,43]. Moreover, phenolic acids such as vanillic acid and protocatechuic acid in GPE exhibited the ability to be anti-inflammatory, and protocatechuic acid can prevent NAFLD by modulating hepatic inflammatory cytokines and gut microbiota [44,45]. In this study, GPE exerted hepatoprotective effects mainly on four aspects: hepatic steatosis, oxidative stress, anti-inflammation, and fibrosis. The preventative therapeutic function of GPE is inseparable from the efficacy of its constituents. In the MCD group, there was a significant decrease in serum levels of TC and TG, while liver levels of TC and TG significantly increased compared with the MCS group, consistent with the phenotype of MCD diet-induced NASH mice. LGPE and HGPE were able to improve oxidative stress by increasing the content of SOD and decreasing the content of MDA, which reduced liver tissue injury. Additionally, both LGPE and HGPE significantly improved the fibrosis of liver tissue. Elevated levels of ALT and AST in the blood specifically signify liver damage. GPE effectively reversed the liver injury in mice with NASH induced by an MCD diet. GPE may thus represent a promising therapeutic agent for the prevention of NASH.

Research has demonstrated that when herbal extracts are ingested, they are usually absorbed directly into human circulation through the intestinal tract, where they are transformed by the intestinal microbiota and exert their effects—either beneficial or detrimental—through the gut–liver axis [46]. Various herbal medicine extracts have exhibited anti-inflammatory ability and an anti-NASH effect by altering the composition of the intestinal microbiota [47,48,49]. In this study, the gut microbiota was modulated by the treatment of GPE. Specifically, *Klebsiella*, which showed a high abundance in the MCD group, was significantly reduced by HGPE. *Klebsiella*, as an opportunistic pathogen, may be one of the causes of NASH [50,51]. There are two mechanisms by which *Klebsiella* promotes the development of NASH. Firstly, *Klebsiella* colonizes the intestine and produces a large amount of endogenous ethanol, which reaches the liver via the portal system, causing mitochondrial dysfunction in hepatocytes and leading to NASH [50]. Secondly, dysregulation of the gut microbiota enhances intestinal permeability. This condition facilitates the leakage of lipopolysaccaride (LPS), produced by *Klebsiella*, into the bloodstream. Subsequently, LPS activates liver macrophages through the portal system, triggering the release of pro-inflammatory cytokines and exacerbating NASH. Serum lipopolysaccharide-binding protein (LBP), an indicator of chronic inflammation, can recognize circulating LPS. In this study, the LBP levels in the blood of mice were significantly decreased in both the LGPE group and the HGPE group. Meanwhile, the relative abundance of *Akkermansia* in the LGPE group appeared to be enriched in comparison to the MCD group. *Akkermansia* is considered a promising candidate for probiotics and has proven effective in improving metabolic modulation, immune regulation, and gut health protection [52]. The current study showed that the symbiotic combination of *Akkermansia* and quercetin ameliorated early obesity and NAFLD by modulating bile acid metabolism and reshaping the microbiota [53]. In summary, GPE remodeled the gut microbiota in NASH mice. LGPE was able to enrich potential probiotics, such as *Akkermansia*, while HGPE was able to inhibit the contents of opportunistic pathogenic bacteria, such as *Klebsiella*.

Toll-like receptor 4 (TLR4) is essential for the functioning of the innate immune system and mediates inflammatory responses through the recognition of LPS or bacterial endotoxins [54]. TLR4 forms a complex with LBP and CD14 to facilitate the binding of LPS [55]. In this experiment, serum LBP levels decreased after GPE administration. With the assistance of CD14, TLR4 triggers the MyD88-dependent pathway [56,57,58,59]. Notably, the MyD88-dependent signaling pathway specifically triggers the activation of genes that are linked to pro-inflammatory mediators, including tumor necrosis factor α (TNF-α) and interleukin 6 (IL-6) [55]. In Kupfer cells, TLR4 triggers the transcription of NF-κB, leading to the release of pro-inflammatory cytokines, including TNF-α and IL-1β [60]. In the current study, HGPE reduced the relative expression of TLR4, CD14, TNF-α, Myd88, and IL-1β in the liver. S. mussotii may mitigate NAFLD by reducing the expression of NF-κB in both the nucleus and the cytoplasm of NAFLD rats [61]. The results indicate that GPE can alter the hepatic transcriptome in NASH mice, potentially alleviating NASH through the aforementioned molecular mechanisms. Furthermore, it has been found that *Akkermansia* may produce a novel tripeptide, Arg-Lys-His, capable of directly binding to TLR4, thereby obstructing TLR4 signal transduction in immune cells. This interaction effectively diminishes the activation of inflammatory cells and the excessive production of pro-inflammatory factors [62]. The results of the IPA analysis revealed that Klebsiella can cause infection and further activates pathogen-associated molecular patterns. Anemoside B4 has been demonstrated to protect against *Klebsiella* pneumoniae-induced pneumonia via the TLR4/Mdy88 signaling pathway in mice [63]. Therefore, it is concluded that GPE may reduce inflammation in the liver through the remodeling of the gut microbiota and the downregulation of inflammatory genes.

Some limitations in the current study include, first, that the experimental design should explore whether GPE affects intestinal barrier function. Second, GPE contains numerous components, and the specific components that actually play a role need to be further identified. Third, to bring the research closer to clinical studies, the next research should use models that more accurately reflect human NASH. In addition, future experiments should focus on the causal relationship between NASH and individual bacteria. Further investigation of these aspects is warranted.

## 5. Conclusions

In conclusion, our research demonstrates that GPE possesses anti-inflammatory and hepatoprotective properties, and its beneficial effects are partly due to gut microbiota modulation. It is important to recognize GPE’s capacity to augment potential probiotics and suppress opportunistic pathogens. This suggests GPE’s viability as a prospective prebiotic agent in the preventive approach to NASH.

## Figures and Tables

**Table 1:** Top 10 compounds in GPE.

Number	Molecular Formula	Molecular Mass	Name	Peak Area	CAS
**20**	C_18_H_30_O_3_	294.22	Hydroxylinolenic acid	2,154,772	1228349-30-1
**7**	C_27_H_30_O_16_	610.15	Rutin	1,745,473	153-18-4
**22**	C_18_H_32_O_3_	296.24	Hydroxylinoleic acid	1,630,636	30207-02-4
**4**	C_8_H_8_O_4_	168.04	Vanillic acid	1,630,182	121-34-6
**9**	C_9_H_10_O_4_	182.06	Methyl vanillate	1,406,646	3943-74-6
**12**	C_15_H_10_O_7_	302.04	Quercetin	1,295,360	117-39-5
**29**	C_35_H_36_N_4_O_5_	592.27	Pheophorbide A	1,293,153	15664-29-6
**1**	C_7_H_6_O_4_	154.03	Protocatechuic acid	1,229,841	99-50-3
**18**	C_27_H_28_N_2_O_4_	444.20	Aurantiamide acetate	845,990	56121-42-7
**15**	C_16_H_12_O_7_	316.06	Isorhamnetin	810,542	480-19-3

## Data Availability

The sequence data presented in this study have been submitted to NCBI SRA database (Accession Number: PRJNA1118223).

## Supplementary Materials

The following supporting information can be downloaded at: https://www.mdpi.com/article/10.3390/nu16111782/s1, Supplementary Table S1 Chemical Composition Information of GPE Based on UPLC-Q-TOF/MS. Supplementary Table S2 Elution gradient. Supplementary Table S3 Mass parameters (Sciex Triple TOF 4600 LC-MS). Supplementary Table S4 The primer sequences for RT-qPCR. Supplementary Figure S1 Differences in CAGs relative abundance between groups.
