# Supplementary material for: Gynostemma pentaphyllum Extract Alleviates NASH in Mice: Exploration of Inflammation and Gut Microbiota
Source: Nutrients. 2024 Jun 6;16(11):1782. doi: 10.3390/nu16111782 (PMC11174846; doi:10.3390/nu16111782)
Supplement: Supplementary file 1 [file nutrients-16-01782-s001.zip › nutrients-2985325-supplementary.pdf]

Supplementary Table S1. Chemical Composition Information of GPE Based on UPLC-Q-TOF/MS

No.	RT (min)	Adduct ions	Measured m/z	Respected m/z	ppm	Formula	M.W.	Identification	MS/MS data	peak area
1	1.48	$[M-H]^-$	153.0206	153.0193	8.3	$C_7H_6O_4$	154.03	Protocatechuic acid	153.0210;109.0303;108.0225;91.0191;81.0349	1229841
2	3.93	$[M+H]^+$	146.0591	146.06	-6.4	C_9H_7NO	145.05	1H-indole-3-aldehyde	146.0597;117.0586;91.0534;77.0378;65.0377	81371
3	7.78	$M+FA-H]^-$	431.1946	431.1923	5.4	$C_{19}H_{30}O_8$	386.19	Roseoside	431.1922;385.1893;223.1362;205.1235;153.0929	39034
4	8.54	$[M-H]^-$	167.0363	167.035	7.9	$C_8H_8O_4$	168.04	Vanillic acid	167.0356;152.0132,108.0229,65.0032	1630182
5	9.28	$[M-H]^-$	195.0304	195.0299	2.6	$C_9H_8O_5$	196.04	5-Formyl-o-vanillic acid	195.0310;167.0359;152.0123;136.0174;108.0224	150267
6	10.29	$[M+H]^+$	197.1174	197.1172	0.9	$C_{11}H_{16}O_3$	196.11	Loliolide	197.1170;179.1064;161.0959;133.1008;105.0698	131711
7	10.71	$[M-H]^-$	609.1505	609.1461	7.2	$C_{27}H_{30}O_{16}$	610.15	Rutin	609.1505;300.0280;271.0258;255.0306	1745473
8	11.29	$[M-H]^-$	463.0922	463.0882	8.6	$C_{21}H_{20}O_{12}$	464.10	Isoquercitrin	463.0931;300.0298;271.0264;255.0313;243.0301	313223
9	12.41	$[M-H]^-$	181.0518	181.0506	6.4	$C_9H_{10}O_4$	182.06	Methyl vanillate	181.0514;153.0199;108.0232	1406646

10	12.72	$[M+NH4]^+$	556.2409	556.2389	3.7	$C_{26}H_{34}O_{12}$	538.21	Olivil 4'-O-glucoside	377.1621;359.1482;329.1401;207.1001	7452
11	13.52	$[M+FA-H]^-$	567.2123	567.2083	7	$C_{26}H_{34}O_{11}$	522.21	Lariciresinol 9-O-β-D-glucopyranoside	341.1429;326.1187;311.0941;179.0577	116056
12	17.96	$[M-H]^-$	301.0359	301.0354	1.7	$C_{15}H_{10}O_7$	302.04	Quercetin	301.0357;273.0413;179.0000;151.0050;121.0309	1295360
13	19.14	$[M-H]^-$	299.0207	299.0197	3.3	$C_{15}H_8O_7$	300.03	Quercetinquinone	299.0207;271.0261;243.0308;231.0304;151.0041	671403
14	20.94	$[M-H]^-$	285.0413	285.0405	2.9	$C_{15}H_{10}O_6$	286.05	Kaempferol	285.0435;257.0137;239.0362;211.0431;169.0671	757915
15	21.53	$[M-H]^-$	315.0525	315.051	4.7	$C_{16}H_{12}O_7$	316.06	Isorhamnetin	315.0521;300.0288;283.0257;271.0265;151.0052	810542
16	23.37	$[M+H]^+$	457.3688	457.3676	2.6	$C_{30}H_{48}O_3$	456.36	Ursolic acid isomer	457.3702;439.3608;421.3488;201.1646;159.1164	468868
17	26.96	$[M-H]^-$	329.0686	329.0667	5.8	$C_{17}H_{14}O_7$	330.07	Ombuin	329.0662;314.0439;299.0207;271.0255;243.0300	351915
18	28.69	$[M+H]^+$	445.2139	445.2122	3.9	$C_{27}H_{28}N_2O_4$	444.20	Aurantiamide acetate	252.1016;224.1067;194.1171;117.0688;105.0325	845990
19	29.94	$[M+FA-H]^-$	721.3685	721.3652	4.6	$C_{33}H_{56}O_{14}$	676.37	Gingerglycolipid A	675.3627;415.1465;397.1357;277.2181	238175
20	30.98	$[M-H]^-$	293.2143	293.2122	7.1	$C_{18}H_{30}O_3$	294.22	Hydroxylinolenic acid	293.2132;275.2028;231.2120;183.1398;171.1032	2154772

21	31.96	$[M+FA-H]^-$	559.315	559.3124	4.7	$C_{27}H_{46}O_9$	514.31	Panaxcerol B	513.3108;277.2197;253.0945	174865
22	32.64	$[M-H]^-$	295.2294	295.2279	5.2	$C_{18}H_{32}O_3$	296.24	Hydroxylinoleic acid	295.2280;277.2175;195.1400;183.1033	1630636
23	33.05	$[M+H]^+$	566.4285	566.4276	1.6	$C_{30}H_{55}N_5O_5$	565.42	Cyclo-(Leu)5	566.4278;453.3443;340.2593;227.1751;199.1798	485826
24	33.88	$[M-H]^-$	555.289	555.2845	8.2	$C_{25}H_{48}O_{11}S$	556.29	1-O-Palmitoyl-3-O-(6-sulfo-6-deoxy-alpha-D-glucopyranosyl)-L-glycerol	555.2853;299.0450;255.2335;225.0078;164.9871	716817
25	36.94	$[M-H]^-$	277.2178	277.2173	1.8	$C_{18}H_{30}O_2$	278.22	Linolenic acid	277.2181;259.2064;233.229	756041
26	38.33	$[M-H]^-$	279.2331	279.233	0.5	$C_{18}H_{32}O_2$	280.24	Linoleic acid	279.2338;261.2251;243.2120	294662
27	38.94	$[M+H]^+$	609.2722	609.2708	2.4	$C_{35}H_{36}N_4O_6$	608.26	10-Hydroxypheophorbide a	609.2748;591.2640;531.2412;485.2335	630107
28	39.54	$[M-H]^-$	255.234	255.233	4.1	$C_{16}H_{32}O_2$	256.24	Palmitic acid	255.2343;237.2262	115297
29	39.55	$[M+H]^+$	593.277	593.2758	1.9	$C_{35}H_{36}N_4O_5$	592.27	Pheophorbide A	593.2771;533.2561;460.2251	1293153
30	41.78	$[M+H]^+$	607.2938	607.2915	3.8	$C_{36}H_{38}N_4O_5$	606.28	Methyl pheophorbide a	607.2939;547.2726;461.2357	337009

Supplementary Table S2

Elution gradient

Time (min)	A%	B%
0～3	5	95
3～13	5～15	95 ~ 85
13～22	15～20	85 ~ 80
22～37	20～28	80 ~ 72
37～52	28～45	72 ~ 55
52～67	45～60	55 ~ 40
67～87	60～95	40 ~ 5
87～89	95	5
89～89.1	95～5	5 ~ 95
89.1～93	5	95

Supplementary Table S3

Mass parameters (Sciex Triple TOF 4600 LC-MS)

MS parameters	Values	MS/MS parameters	Values
TOF mass range	50～1700	MS/MS mass range	50～1250
Ion Source Gas 1（psi）	50	Declustering Potential（V）	100
Ion Source Gas 2（psi）	50	Collision Energy（eV）	±40
Curtain Gas（psi）	35	Collision Energy Spread（eV）	20
Ion Spray Voltage Floating (V)	-4500/5000	Ion Release Delay（ms）	30
Ion Source Temperature (°C)	500	Ion Release Width（ms）	15
Declustering Potential（V）	100		
Collision Energy（eV）	10		

Supplementary Table S4. Primer sequences for RT-qPCR

Gene	Forward	Reverse
TLR4	TGTTCTTCTCCTGCCTGACA	CATCAGGGACTTTGCTGAGTT
CD14	GAGTTGTGACTGGCCCAGTCAGC	GCAAAAGCCAGAGTTCCTGAC
TNF-α	TAGCCAGGAGGGAGAACAGA	TTTTCTGGAGGGAGATGTGG
Myd88	AGGACAAACGCCGGAACTTT	AGGACAAACGCCGGAACTTT
IL-1β	GAAATGCCACCTTTTGACAGTG	CTGGATGCTCTCATCAGGACA
CCR5	AGCCTGATCCTGCCTCTACTTGTC	GCCCTGTGCCTCTTCTTCTCATTC
GAPDH	TGTTTCCTCGTCCCGTAG	CAATCTCCACTTTGCCACT

Supplementary Figure S1. Differences in CAGs relative abundance between groups

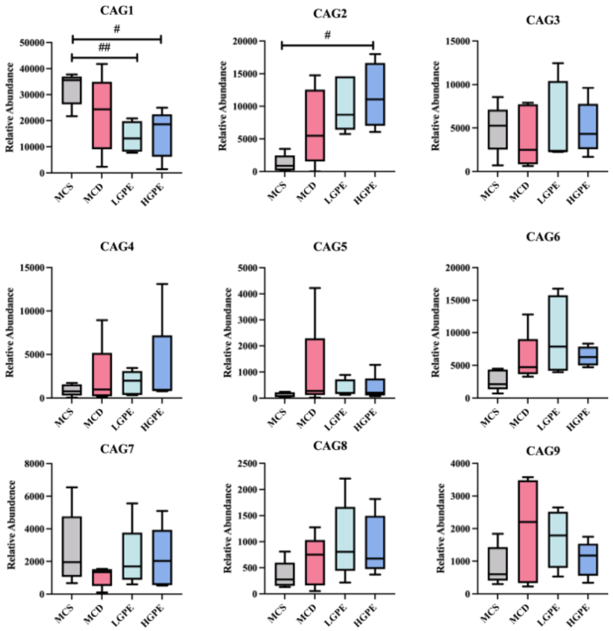

Supplementary Figure S1: Group-level abundance differentiation of CAGs. Data are visualized by box-plot. Box represents the interquartile range.

The line inside the box represents the median. And whiskers denote the minimum and maximum value. $^{\#}P < 0.05$, $^{\#\#}P < 0.01$ vs MCS (n=5)
